# Restoration of Pattern Recognition Receptor Costimulation to Treat Chromoblastomycosis, a Chronic Fungal Infection of the Skin

**DOI:** 10.1016/j.chom.2011.04.005

**Published:** 2011-05-19

**Authors:** Maria da Glória Sousa, Delyth M. Reid, Edina Schweighoffer, Victor Tybulewicz, Jürgen Ruland, Jean Langhorne, Sho Yamasaki, Philip R. Taylor, Sandro R. Almeida, Gordon D. Brown

**Affiliations:** 1 Aberdeen Fungal Group, Section of Immunology and Infection, Institute of Medical Sciences, University of Aberdeen, Aberdeen AB25 2ZD, UK; 2 National Institute for Medical Research, London NW7 1AA, UK; 3 Institut für Molekulare Immunologie, Klinikum rechts der Isar, Technische Universität München, 81675 Munich, Germany; 4 Laboratory of Signaling in the Immune System, Helmholtz Zentrum München - German Research Center for Environmental Health, 85764 Neuherberg, Germany; 5 Division of Molecular Immunology, Medical Institute of Bioregulation, Kyushu University, Fukuoka 812-8582, Japan; 6 Department of Infection, Immunity, and Biochemistry, School of Medicine, Cardiff University, Cardiff CF14 4XN, Wales, UK; 7 Departamento de Análises Clínicas e Toxicológicas, Faculdade de Ciências Farmacêuticas, Universidade de São Paulo, São Paulo 05508-900, Brazil

## Abstract

Chromoblastomycosis is a chronic skin infection caused by the fungus *Fonsecaea pedrosoi*. Exploring the reasons underlying the chronic nature of *F. pedrosoi* infection in a murine model of chromoblastomycosis, we find that chronicity develops due to a lack of pattern recognition receptor (PRR) costimulation. *F. pedrosoi* was recognized primarily by C-type lectin receptors (CLRs), but not by Toll-like receptors (TLRs), which resulted in the defective induction of proinflammatory cytokines. Inflammatory responses to *F. pedrosoi* could be reinstated by TLR costimulation, but also required the CLR Mincle and signaling via the Syk/CARD9 pathway. Importantly, exogenously administering TLR ligands helped clear *F. pedrosoi* infection in vivo. These results demonstrate how a failure in innate recognition can result in chronic infection, highlight the importance of coordinated PRR signaling, and provide proof of the principle that exogenously applied PRR agonists can be used therapeutically.

## Graphical abstract

**Graphical Abstract:**

**Figure:**
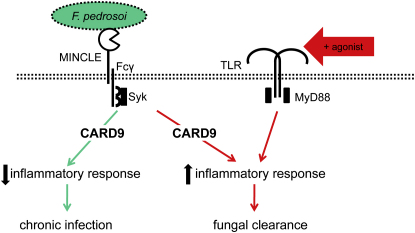

**Highlights:**

► *F. pedrosoi* is recognized by C-type lectins, but not by Toll-like receptors ► Inflammatory responses to *F. pedrosoi* are defective, which leads to chronic infection ► Exogenous TLR costimulation restores inflammatory responses and clears infection

## Introduction

Chromoblastomycosis is a chronic nonfatal mycosis involving the skin and subcutaneous tissues, which is caused by a number of melanized fungi. The disease occurs worldwide, but is observed most frequently in tropical and subtropical regions of Africa and Latin America (Ameen, 2009; Santos et al., 2007). Infection is acquired by the accidental inoculation of the etiologic agent into the subcutaneous tissues, but it usually takes decades following inoculation before clinical symptoms develop. The infection is characterized by erythematous papules, which develop with varying morphology, and systemic invasion is rare (Ameen, 2009; Santos et al., 2007). There are no standard treatments, although approaches usually include chemotherapy, multiple surgical excisions, and/or cryosurgery with liquid nitrogen. Furthermore, there is often a poor response to oral antifungal drugs, and most attempts at treatment have only a modest success rate (Ameen, 2009; Santos et al., 2007).

A number of melanized dematiaceous fungi have been associated with chromoblastomycosis, but the most common agent causing this disease is *Fonsecaea pedrosoi* (Bonifaz et al., 2001). Little is known about this fungus, its cell wall structure, how it is recognized by the host, or the protective /nonprotective immune responses that are triggered upon infection. Host defense against experimental chromoblastomycosis has been shown to rely mainly on the ingestion and elimination of fungal cells by cells of the innate immune system, especially neutrophils and macrophages, but there is also some evidence supporting a requirement of CD4^+^, although not CD8^+^, T cell-mediated immune responses (Ameen, 2009; Santos et al., 2007). Notable features of patients with chromoblastomycosis include increased IL-10 and low levels of IFN-γ (Ameen, 2009; Santos et al., 2007).

We were interested in understanding the reasons underlying the chronic nature of infection with *F. pedrosoi* and wanted to explore the possibility that the chronicity of this infection might stem from an inappropriate innate immune response. In this report, we show that *F. pedrosoi* is recognized by C-type lectin receptors (CLRs), but that there is a lack of sufficient costimulation of the Toll-like receptors (TLRs), and that this results in defective inflammatory responses. Excitingly, we could re-establish this costimulatory cytokine response by exogenous administration of TLR agonists, which could also be used to resolve the infection in vivo.

## Results

### Establishment and Characterization of a Murine Model of Chromoblastomycosis

**Figure 1:**
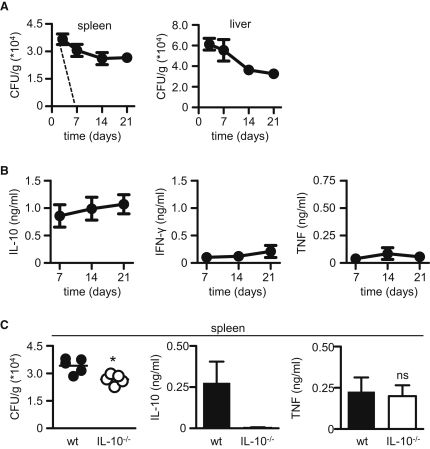
Establishment of a Murine Model of Infection with *F. pedrosoi* (A) Fungal burdens in the spleen and liver (black lines) at various time points, as indicated, following i.p. infection with *F. pedrosoi* conidia. Shown for comparison is the clearance rate of a similar dose of *C. albicans* (dotted line) (Tsoni et al., 2009). (B) Characterization of the levels of IL-10, IFN-γ, and TNF from infected spleens. (C) Characterization of fungal burdens and cytokine levels in the spleens of wild-type (WT; black symbols) versus IL-10^−/−^ (white symbols) mice at day 7 postinfection. See also Figure S1. Values shown are the mean ± SEM of two pooled experiments, except for (C), which is mean ± SD. ^∗^p < 0.05.

To explore the reasons underlying the chronicity of chromoblastomycosis, we made use of an established murine model (Cardona-Castro and Agudelo-Flórez, 1999), where mice are infected intraperitoneally (i.p.) with *F. pedrosoi* conidia, following which the organism disseminates to the liver and spleen, where it persists for many weeks (Figure 1A). By comparison, infection with a pathogen causing an acute infection, such as *Candida albicans*, is cleared rapidly (Tsoni et al., 2009) (dotted line in Figure 1A). Characterization of the spleens of the infected animals revealed high levels of IL-10 as well as low levels of TNF and IFN-γ, cytokine profiles similar to those described in infected humans (Ameen, 2009; Santos et al., 2007) (Figure 1B).

To determine whether IL-10 was contributing to the persistence of the infection in our mice, we compared fungal burdens and cytokine profiles in wild-type and IL-10-deficient animals 7 days after infection. This time point was chosen as it allowed us to examine primarily the innate response to this pathogen (see later). Unexpectedly, the lack of IL-10 only marginally reduced the fungal burdens in the organs of the infected animals and did not greatly influence the inflammatory responses to this pathogen, as determined by measuring the levels of TNF in the infected organs (Figures 1C and S1). Thus, these results suggested that IL-10 was not a major factor involved in establishing the chronicity of infection with *F. pedrosoi*.

### *F. pedrosoi* Fails to Induce Inflammatory Responses from Macrophages

**Figure 2:**
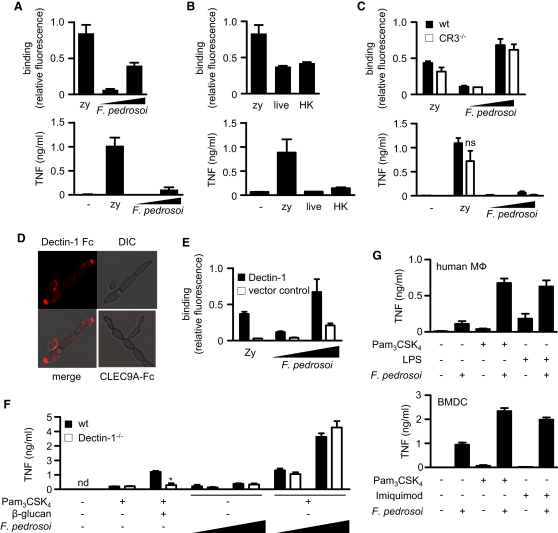
The Response of Thioglycollate-Elicited Peritoneal Macrophages to *F. pedrosoi* Is Defective, but Can Be Restored Following Exogenous TLR Costimulation (A) Binding of *F. pedrosoi* and measurement of TNF responses in macrophage culture supernatants, 3 hr after stimulation with conidia (moi 5:1 and 50:1). (B) Binding of live and heat-killed (HK) *F. pedrosoi* and measurement of TNF responses in macrophage culture supernatants, 3 hr after stimulation with conidia (MOI 5:1). (C) Binding of *F. pedrosoi* and measurement of TNF responses in wild-type (WT; black bars) versus CR3^−/−^ (white bars) macrophage culture supernatants, 3 hr after stimulation with conidia (MOI 5:1 and 50:1). (D) Confocal image showing staining of live *F. pedrosoi* conidia and hyphae with soluble Fc-Dectin-1 or Fc-CLEC9A chimeric proteins. See also Figure S2A. (E) Binding of *F. pedrosoi* conidia (MOI 1:1 and 1:10) to RAW264.7 cells transduced with Dectin-1 (black bars) or vector control (white bars). (F) Costimulation of wild-type (WT; black bars) or Dectin-1^−/−^ (white bars) thioglycollate-elicited macrophages with Pam_3_CSK_4_, β-glucan particles, or *F. pedrosoi* (MOI 5:1 and 25:1), as indicated. See also Figures S2C and S2D. (G) Costimulation of human monocyte-derived macrophages (MΦ) or murine bone-marrow-derived dendritic cells (BMDCs) with *F. pedrosoi* (MOI 5:1), Pam_3_CSK_4_ (10 ng/ml), LPS (1 ng/ml), or Imiquimod (1μg/ml), as indicated. See also Figure S2B. In some experiments, zymosan (zy; 25 particles per cell) was included as a positive control. Values shown are the mean ± SD, and the data are representative of at least two independent experiments, except for the human MΦ, which are mean ± SEM of pooled data from three donors. ^∗^p < 0.05.

We next explored the innate recognition of *F. pedrosoi* by characterizing its interactions with thioglycollate-elicited macrophages in vitro. We found that the conidia bound to macrophages in a dose-dependent fashion, yet surprisingly failed to stimulate the production of TNF, even at high multiplicities of infection (moi) (Figure 2A). In contrast, recognition of the fungal particle zymosan, which was included as a control in these experiments, induced a robust inflammatory response, as expected (Brown et al., 2003).

To explore the possibility that *F. pedrosoi* was actively suppressing leukocyte inflammatory responses, we examined the interaction of macrophages with heat-killed (HK) conidia. However, despite the heat treatment, macrophages still failed to respond to the conidia (Figure 2B). In addition, prolonged coculture for 20 hr with viable conidia, during which time the pathogen formed hyphae, similarly did not result in the induction of TNF (data not shown). The targeting of complement receptor 3 (CR3) is another suppressive mechanism of fungi (Brandhorst et al., 2004); however, loss of CR3 did not restore TNF responses to *F. pedrosoi* conidia (Figure 2C). Thus, the lack of inflammatory responses to *F. pedrosoi* was due to a failure of innate recognition and not active suppression by the pathogen.

### Inflammatory Responses to *F. pedrosoi* Can Be Reinstated by TLR Costimulation

We have previously demonstrated that the recognition of fungal β-glucans by macrophages is not sufficient to induce inflammatory responses, in the absence of additional costimulation through MyD88-coupled TLRs (Brown et al., 2003; Dennehy et al., 2008; Rosas et al., 2008). Thus, we next considered the possibility that the failure of *F. pedrosoi* conidia to induce inflammatory responses was due to a lack of PRR costimulation. We first determined whether β-glucans were exposed in *F. pedrosoi* and could demonstrate that both conidial and hyphal forms of this pathogen displayed these carbohydrates at the cell surface (Figure 2D). Interestingly, heat killing did not significantly alter the level of β-glucan exposure (Figure S2A). Using transfected macrophages, we could also demonstrate that the β-glucan receptor Dectin-1 could directly mediate the binding of *F. pedrosoi* conidia to host cells (Figure 2E).

We then explored the effect of TLR costimulation on macrophage inflammatory responses by making use of substimulatory doses of the TLR2 agonist Pam_3_CSK_4_, which we had previously shown to work synergistically with Dectin-1 (Dennehy et al., 2008). Remarkably, while the addition of *F. pedrosoi* conidia to macrophages failed to induce significant levels of TNF, as we had observed before, costimulation with Pam_3_CSK_4_ induced robust responses (Figure 2F). Similar results were also observed using human peripheral blood-derived macrophages and murine bone-marrow-derived dendritic cells (BMDCs) (Figure 2G). Furthermore, as we had previously demonstrated with purified β-glucan (Dennehy et al., 2008), costimulation with *F. pedrosoi* could be achieved using multiple TLR agonists, including LPS (TLR4) and Imiquimod (TLR7) (Figures 2G and S2B). It is notable that the stimulation of BMDCs with *F. pedrosoi* alone induced some TNF, which was expected, as stimulation of the Dectin-1/Syk signaling pathway in dendritic cells (DCs) is known to be sufficient for cytokine induction (Dennehy et al., 2009; Rogers et al., 2005; Rosas et al., 2008).Thus, the inability of *F. pedrosoi* to induce robust inflammatory responses was due to a lack of costimulation of the TLR pathway.

To confirm the role of Dectin-1 in these responses, we next assessed the costimulatory response in Dectin-1-deficient macrophages. As shown previously (Dennehy et al., 2008), the costimulatory response to highly purified β-glucans was ablated in Dectin-1^−/−^ cells. Unexpectedly, Dectin-1-deficient macrophages displayed no alterations in their ability to induce costimulatory responses to *F. pedrosoi* conidia (Figure 2F). Furthermore, we found that loss of Dectin-1 had no effect on the ability of primary macrophages to bind *F. pedrosoi* conidia (Figure S2C), and characterization of Dectin-1^−/−^ mice revealed only marginal effects on fungal burdens in the organs of the infected animals (Figure S2D). Thus, Dectin-1 does not contribute to the costimulatory responses that were observed in vitro and plays only a minor role during infection in vivo.

### *F. pedrosoi*-TLR Costimulation Requires Mincle and Signaling through the Syk/CARD9 Pathway

**Figure 3:**
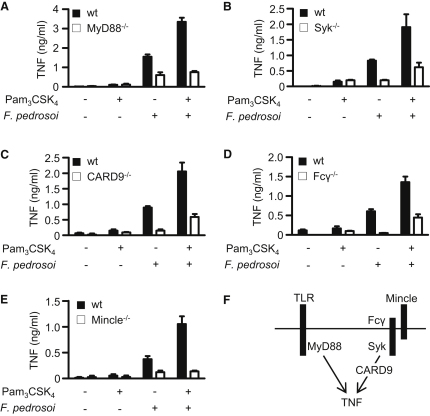
Characterization of the Components Involved in the TLR/*F. pedrosoi* Costimulatory Response (A–E) Measurement of TNF levels in BMDC supernatants following a 3 hr stimulation with Pam_3_CSK_4_ or *F. pedrosoi* (MOI 5:1), as indicated. Responses were measured in wild-type (WT; black bars) cells versus BMDCs deficient (white bars) in MyD88 (A), Syk (B), CARD9 (C), FcRγ (D), or Mincle (E). See also Figure S3. Values shown are the mean ± SD, and the data are representative of at least two independent experiments. (F) Cartoon representation of the costimulatory signaling pathway induced by *F. pedrosoi* and exogenous TLR costimulation.

To determine the signaling pathways and receptors involved in mediating the costimulatory response, we next characterized BMDCs deficient in various signaling molecules. We first examined BMDCs deficient in MyD88 to confirm the involvement of this pathway and, as expected, found that the coaddition of *F. pedrosoi* and Pam_3_CSK_4_ failed to induce a robust inflammatory response in these cells (Figure 3A). Interestingly, the response to *F. pedrosoi* alone was also partly attenuated, indicating some involvement of the MyD88 pathway in sensing of this pathogen by DCs. Indeed when examined further, we found that mice deficient in MyD88 had enhanced fungal burdens during infection (Figure S3A). We next determined if the costimulatory response required signaling through Syk and CARD9, as we had shown for Dectin-1 (Dennehy et al., 2008), and observed that BMDCs deficient in Syk (Figure 3B) or CARD9 (Figure 3C) failed to induce robust inflammatory responses in the presence of *F. pedrosoi* and Pam_3_CSK_4_. The responses to *F. pedrosoi* alone were also attenuated in these cells.

To date, the only other Syk-coupled receptors that have been implicated in fungal recognition are the CLRs Mincle and Dectin-2, and both signal through the FcRγ chain (Drummond et al., 2011). Hence, we examined the role of this signaling adaptor and found that the costimulatory response was lost in Fcγ^−/−^ BMDCs (Figure 3D). We then explored the possibility of Dectin-2 involvement, by inhibiting this receptor with blocking monoclonal antibodies, but observed no effect on these responses (Figure S3B). In contrast, we found that the costimulatory responses were completely ablated in Mincle^−/−^ BMDCs and that this defect was specific for the recognition of *F. pedrosoi*, as Mincle^−/−^ BMDCs retained normal responses to LPS (Figures 3E and S3C). Thus, these results identify the Fcγ-coupled CLR Mincle as a major receptor involved in the innate recognition of *F. pedrosoi*. A model of the proposed costimulatory pathway is shown in Figure 3F.

### TLR Costimulation Cures *F. pedrosoi* Infection

**Figure 4:**
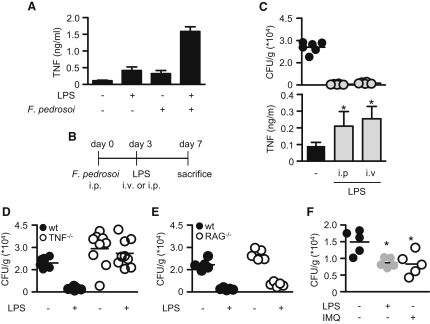
Curing *F. pedrosoi* Infection In Vivo by Exogenous Administration of TLR Agonists (A) TNF in the peritoneum of mice 3 hr after the i.p. administration of *F. pedrosoi* or LPS (10 ng), as indicated. Values shown are the mean ± SEM of two pooled experiments. (B) Schematic diagram of the experimental approach, showing i.p. infection with *F. pedrosoi,* followed by i.p. or i.v. injection on day 3 with a single low dose of LPS (10 ng). The mice were sacrificed and analyzed 4 days later (day 7). (C) Fungal burdens and TNF levels in the spleens of infected mice with or without LPS treatment, as indicated. See also Figure S4A. Values shown are the mean ± SD. ^∗^p < 0.05. (D) Fungal burdens in the spleens of infected wild-type (black circles) or TNF^−/−^ (white circles) mice with or without LPS treatment, as indicated. See also Figure S4B. (E) Fungal burdens in the spleens of infected wild-type (black circles) or RAG2^−/−^ (white circles) mice with or without LPS treatment, as indicated. See also Figure S4C. (F) Fungal burdens in the footpads at day 7 of untreated infected mice (black circles), i.p. LPS-treated mice (gray circles), or mice treated daily with a topical application of Imiquimod (IMQ) (white circles).

The failure of *F. pedrosoi* to induce robust inflammatory responses provides a possible explanation for the persistence of this pathogen, so we investigated the possibility that artificial costimulation of the TLR pathway, to reinstate inflammatory responses, would help resolve the infection in vivo. We first determined if this was possible by administering *F. pedrosoi* i.p., with or without LPS, and monitoring TNF production in the peritoneal cavity after 3 hr. LPS was chosen for these and subsequent experiments, as the effects and dosage of this TLR agonist in vivo are well characterized and as we had shown it to be able to costimulate inflammatory responses to *F. pedrosoi* in vitro (Dennehy et al., 2008) (see Figures 2G and S2B). As shown in Figure 4A, the administration of either *F. pedrosoi* conidia or a low dose of LPS induced little TNF, but when added in combination, they induced a robust inflammatory response.

To demonstrate an effect on fungal clearance, mice were infected with *F. pedrosoi* and the disease was allowed to establish for 3 days, following which a single low dose of LPS was administered either i.p. or i.v., and the infection in the organs was assessed after a further 4 days (Figure 4B). Remarkably, the administration of a single low dose of LPS, via either route, resulted in a near complete clearance of the pathogen from both the spleen and liver, in comparison to the untreated animals (Figures 4C and S4A). Furthermore, as predicted from our in vitro analyses, the administration of LPS significantly increased the levels of TNF in the infected organs (Figures 4C and S4A). This induction of TNF was critical for fungal clearance, since we found LPS to have no beneficial effect in mice deficient in this cytokine (Figures 4D and S4B). In addition, similar effects of LPS on fungal burdens were observed in RAG2^−/−^ mice (Figures 4E and S4C).

Our results suggest that the administration of TLR agonists may be a form of treatment for chromoblastomycosis in humans, ideally through the topical application of these agonists to infected skin. However, it is possible that TLR agonists would not be effective in treating subcutaneous infections. To explore this, we infected mice subcutaneously with *F. pedrosoi* and then treated some animals with topical applications of the FDA-approved agonist Imiquimod, which was also capable of inducing costimulatory activity in vitro (see Figure 2). Remarkably, we found that the topical application of Imiquimod significantly reduced fungal burdens in the skin at day 7 postinfection (Figure 4F). Similar reductions in fungal burdens were also obtained in these tissues following the i.p. administration of LPS on day 3 postinfection. Thus, we conclude that reinstating innate inflammatory responses to *F. pedrosoi* by artificial TLR costimulation in vivo can help resolve this normally persistent infection.

## Discussion

Very little is known about the immunology underlying chromoblastomycosis or the reasons for the chronicity of the disease. In this report we demonstrate that the chronic nature of this infection stems from inadequate innate recognition and the subsequent failure to mount protective inflammatory responses. This failure was not due to active suppression by *F. pedrosoi*, as inflammatory responses to HK organisms were similarly defective. Furthermore, this chronicity was not due to high levels of IL-10, as the level of infection was not greatly altered in mice deficient in this immunosuppressive cytokine. Although *F. pedrosoi* was recognized by leukocyte CLRs, particularly Mincle, we found that this recognition was not sufficient in and of itself to trigger protective inflammatory responses.

These findings were reminiscent of β-glucan recognition by Dectin-1, which required costimulation of MyD88-coupled TLRs to induce robust inflammatory responses (Dennehy et al., 2008). Indeed, we found that costimulation of leukocytes with purified TLR agonists induced robust inflammatory responses to *F. pedrosoi*, indicating that it was the lack of recognition by this PRR family that was responsible for the defective innate responses. Like Dectin-1, the costimulatory response required signaling via the Syk-CARD9 pathway, but was triggered by the FcRγ-coupled CLR Mincle. We could demonstrate this definitively by showing that the costimulatory responses to this organism were lost in cells deficient in any of these signaling components. While it is likely that Mincle is recognizing a mannose-based cell wall component (Yamasaki et al., 2009), the identity of the ligand is unclear. It should also be noted that unlike macrophages, the recognition of *F. pedrosoi* did induce some response in DCs, even in the absence of TLR costimulation. For other Syk-dependent responses, such as those triggered through Dectin-1, these cellular differences have been linked to the effects of cytokines, such as the GM-CSF used to generate the DCs and the differential usage of CARD9, but the underlying reasons are still not fully understood (Drummond et al., 2011). Nonetheless, as for Dectin-1, TLR costimulation induced robust inflammatory responses to *F. pedrosoi* in both cell types.

The immunostimulatory components within the cell walls of many fungi are thought to be shielded, possibly providing an explanation for the failure of *F. pedrosoi* to induce protective responses. However, the lack of response to dead organisms indicates that this is unlikely, as heat killing is thought to disrupt the cell wall architecture and expose underlying PAMPs (Robinson et al., 2009). Although this suggests that *F. pedrosoi* lacks sufficient levels of exposed TLR ligands to stimulate robust responses, the partial ablation of TNF production in the MyD88^−/−^ DCs does indicate the presence of some ligands for these receptors. What the nature of these ligands may be is unclear, as the architecture and composition of the cell wall of *F. pedrosoi* is largely unknown. Furthermore, the significant increase in fungal burdens in the MyD88-deficient mice might also suggest some contribution of TLR recognition to the control of this pathogen, but the interpretation of these results are complicated by the role of MyD88 in IL-1 receptor signaling and the importance of IL-1 signaling in antifungal immunity.

The failure of leukocytes to induce robust inflammatory responses to *F. pedrosoi* led us to test the possibility that inducing these responses, through the exogenous administration of TLR agonists, could help resolve the infection in vivo. Indeed, using a murine model of systemic infection, we found that the administration of LPS, either i.v. or i.p., significantly reduced fungal burdens in infected organs. This increased fungal clearance was due to the enhanced inflammatory responses triggered by the exogenous costimulation of the TLRs, as LPS failed to have an effect in TNF^−/−^ mice. However, LPS has also been shown to induce Mincle expression in vitro (Matsumoto et al., 1999), therefore raising the possibility of an indirect contribution of this TLR agonist in host responses.

While the systemic mouse model of chromoblastomycosis is not an accurate representation of the human subcutaneous infection, it is thought to be the best model for studying the chronic nature of this disease (Cardona-Castro and Agudelo-Flórez, 1999). Indeed, the systemic infection is chronic in mice, and the pathogen persists for many weeks in the organs of untreated animals. Importantly, the ability of artificial TLR costimulation to restore inflammatory responses and induce rapid fungal clearance, particularly in RAG-deficient mice, clearly demonstrates that the persistence of this fungal pathogen is primarily due to defective innate recognition. However, chronic infection with *F. pedrosoi* also leads to dysregulated adaptive immunity (Mazo Fávero Gimenes et al., 2005), which was not addressed here, and it would be interesting to examine the effect of TLR agonist treatment on the development of these responses during the infection.

Excitingly, these results suggest that the exogenous administration of TLR agonists could be used to treat human patients. Although the majority of our studies were performed using disseminated infections, we could demonstrate that a similar approach was also feasible in a subcutaneous infection model. While this approach requires further optimization, we found that the topical application of Imiquimod significantly reduced fungal burdens in the infected tissues. Our in vitro data also suggest that such an approach may work in humans, as human macrophages, like their mouse counterparts, failed to induce robust inflammatory responses to *F. pedrosoi* in the absence of artificial TLR costimulation.

In conclusion, we have shown that the persistence of infection with *F. pedrosoi* is due to a failure in innate recognition, stemming from a lack of TLR costimulation. It is tempting to speculate that defective PRR costimulation may underlie the development of other chronic infections and that enhancement of inflammatory responses may be responsible for the anti-infective activities reported for many immunostimulants, such as β-glucan. Our results also highlight the importance of coordinated PRR signaling and demonstrate how exogenously applied PRR agonists could be used to treat these types of diseases. For chromoblastomycosis in humans, this might simply involve the topical application of appropriate TLR ligands, such as Imiquimod.

## Experimental Procedures

### Animals

Male or female 8- to 14-week-old 129Sv, 129Sv Dectin-1^−/−^ (Taylor et al., 2007), BALB/C, BALB/C IL-10^−/−^ (Dewals et al., 2010), C57BL/6, C57BL/6 RAG2^−/−^ (Dewals et al., 2010), C57BL/6 TNF^−/−^ (Marino et al., 1997), and C57BL/6 Myd88^−/−^ (Fremond et al., 2004) mice were obtained from the specific pathogen-free facility of the University of Cape Town. All animal experimentation was performed using groups of 5–10 animals, repeated at least once, and conformed to institutional guidelines for animal care and welfare.

### *F. pedrosoi* Growth Conditions and Fluorescent Labeling

*F. pedrosoi* ATCC 46428 was streaked onto potato dextrose agar or Sabouraud agar plates for isolation of individual colonies for 12 days. Colonies were cultured in a shaking incubator for 72 hr at 30°C in potato broth for in vitro and in vivo assays. The conidia were filtered to remove hyphae and washed with PBS before use (live conidia) or were heat killed by boiling for 30 min. For fluorescence labeling, washed live or HK conidia were labeled with Rhodamine Green-X (Invitrogen) (200 μg/ml) for 30 min at 25°C, followed by extensive washing. To detect surface-exposed β-glucans, washed live or HK *F. pedrosoi* cells were stained with soluble Fc-Dectin-1(Graham et al., 2006) or Fc-CLEC9A (Huysamen et al., 2008) chimeric proteins (5 μg/ml), as described previously (Graham et al., 2006). In some experiments, soluble β-glucan (100 μg/ml) was mixed with Fc-Dectin-1 for 30 min prior to staining.

### Cells and In Vitro Fungal Stimulations

DCs were generated from Syk^−/−^ fetal livers, as described (Rogers et al., 2005), or from the bone marrow of CARD9^−/−^ (Gross et al., 2009), FcRγ^−/−^ (Takai et al., 1994), Mincle^−/−^ (Yamasaki et al., 2009), MyD88, and C57BL/6 mice, using standard protocols. Human monocyte-derived macrophages and thioglycollate-elicited macrophages were generated as described previously (Dennehy et al., 2008; Willment et al., 2003). Macrophages and BMDCs were plated the night before use in 24-well plates at a density of 2.5 × 10^5^ cells per well in RPMI medium with 10% heat-inactivated FCS. The RAW264.7 macrophages expressing Dectin-1 (Brown et al., 2002) and control cells were plated at 2.5 × 10^5^ cells per well in medium containing 0.4 mg/ml G418 (Invitrogen).

For the in vitro binding and cytokine assays, unlabeled or Rhodamine Green-X-labeled live or HK *F. pedrosoi* were added to the cells, as indicated, and incubated for 30 min at 37°C. In some experiments, the following compounds were also added alone or in combination, as indicated: unlabeled or FITC-labeled zymosan (25 particles per cell) (Invitrogen), purified β-glucan particles (100 μg/ml) (Dennehy et al., 2008), Pam_3_CSK_4_ (10 ng/ml) (Invivogen), LPS (1 ng/ml) (Sigma), and Imiquimod (1 μg/ml) (Invivogen). Unbound particles were removed by washing. The medium was replaced, and the cells were cultured for a further 3 hr for analysis of TNF by ELISA (BD Biosciences). Cytokine stimulations were not influenced by the presence or absence of a fluorescent label on the fungal particles (data not shown). After incubation, supernatants were stored at −80°C until use, cells were lysed in 3% (volume/volume) Triton X-100, and fluorescence was measured with a Titertek Fluoroskan II (Labsystems).

### In Vivo Models

For in vivo infections, mice were infected with 2 × 10^6^ conidia of *F. pedrosoi* i.p. In some experiments, LPS (10 ng) (Sigma) was also administered to mice i.p. or i.v., 3 days after infection with *F. pedrosoi*. At appropriate time points after infection, as indicated in the text, the animals were sacrificed, and colony-forming units were determined in disaggregated whole livers and spleens by serial dilution onto Sabouraud agar plates. Organ cytokine levels were determined by ELISA (BD Biosciences). To measure peritoneal inflammation, mice were injected i.p. with 2 × 10^6^ live *F. pedrosoi* and/or LPS (10 ng/ml) and were killed after 3 hr. TNF was measured in peritoneal lavage fluid by ELISA (BD Biosciences). For infection of the footpad, mice were subcutaneously injected with 1 × 10^7^ *F. pedrosoi* conidia, and then 5% Imiquimod cream (Aldara, Graceway Phamaceuticals) was topically applied daily to the site of infection, where indicated. In other mice, 100 ng of LPS was administered i.p. at day 3 after infection. All mice were sacrificed at day 7, and colony-forming units were determined in disaggregated footpads by serial dilution onto Sabouraud agar plates.

### Statistics

Student's t test was used for the analysis of two groups. Results were considered statistically significant with p values of ≤ 0.05.

## Supplemental Information

Document S1. Four Figures

## References

[bib1] Ameen M. (2009). Chromoblastomycosis: clinical presentation and management. Clin. Exp. Dermatol..

[bib2] Bonifaz A., Carrasco-Gerard E., Saúl A. (2001). Chromoblastomycosis: clinical and mycologic experience of 51 cases. Mycoses.

[bib3] Brandhorst T.T., Wüthrich M., Finkel-Jimenez B., Warner T., Klein B.S. (2004). Exploiting type 3 complement receptor for TNF-alpha suppression, immune evasion, and progressive pulmonary fungal infection. J. Immunol..

[bib4] Brown G.D., Taylor P.R., Reid D.M., Willment J.A., Williams D.L., Martinez-Pomares L., Wong S.Y., Gordon S. (2002). Dectin-1 is a major beta-glucan receptor on macrophages. J. Exp. Med..

[bib5] Brown G.D., Herre J., Williams D.L., Willment J.A., Marshall A.S.J., Gordon S. (2003). Dectin-1 mediates the biological effects of beta-glucans. J. Exp. Med..

[bib6] Cardona-Castro N., Agudelo-Flórez P. (1999). Development of a chronic chromoblastomycosis model in immunocompetent mice. Med. Mycol..

[bib7] Dennehy K.M., Ferwerda G., Faro-Trindade I., Pyz E., Willment J.A., Taylor P.R., Kerrigan A., Tsoni S.V., Gordon S., Meyer-Wentrup F. (2008). Syk kinase is required for collaborative cytokine production induced through Dectin-1 and Toll-like receptors. Eur. J. Immunol..

[bib8] Dennehy K.M., Willment J.A., Williams D.L., Brown G.D. (2009). Reciprocal regulation of IL-23 and IL-12 following co-activation of Dectin-1 and TLR signaling pathways. Eur. J. Immunol..

[bib9] Dewals B., Hoving J.C., Horsnell W.G., Brombacher F. (2010). Control of Schistosoma mansoni egg-induced inflammation by IL-4-responsive CD4(+)CD25(-)CD103(+)Foxp3(-) cells is IL-10-dependent. Eur. J. Immunol..

[bib10] Drummond R.A., Saijo S., Iwakura Y., Brown G.D. (2011). The role of Syk/CARD9 coupled C-type lectins in antifungal immunity. Eur. J. Immunol..

[bib11] Fremond C.M., Yeremeev V., Nicolle D.M., Jacobs M., Quesniaux V.F., Ryffel B. (2004). Fatal Mycobacterium tuberculosis infection despite adaptive immune response in the absence of MyD88. J. Clin. Invest..

[bib12] Graham L.M., Tsoni S.V., Willment J.A., Williams D.L., Taylor P.R., Gordon S., Dennehy K., Brown G.D. (2006). Soluble Dectin-1 as a tool to detect beta-glucans. J. Immunol. Methods.

[bib13] Gross O., Poeck H., Bscheider M., Dostert C., Hannesschläger N., Endres S., Hartmann G., Tardivel A., Schweighoffer E., Tybulewicz V. (2009). Syk kinase signalling couples to the Nlrp3 inflammasome for anti-fungal host defence. Nature.

[bib14] Huysamen C., Willment J.A., Dennehy K.M., Brown G.D. (2008). CLEC9A is a novel activation C-type lectin-like receptor expressed on BDCA3+ dendritic cells and a subset of monocytes. J. Biol. Chem..

[bib15] Marino M.W., Dunn A., Grail D., Inglese M., Noguchi Y., Richards E., Jungbluth A., Wada H., Moore M., Williamson B. (1997). Characterization of tumor necrosis factor-deficient mice. Proc. Natl. Acad. Sci. USA.

[bib16] Matsumoto M., Tanaka T., Kaisho T., Sanjo H., Copeland N.G., Gilbert D.J., Jenkins N.A., Akira S. (1999). A novel LPS-inducible C-type lectin is a transcriptional target of NF-IL6 in macrophages. J. Immunol..

[bib17] Mazo Fávero Gimenes V., Da Glória de Souza M., Ferreira K.S., Marques S.G., Gonçalves A.G., Vagner de Castro Lima Santos D., Pedroso e Silva Cde.M., Almeida S.R. (2005). Cytokines and lymphocyte proliferation in patients with different clinical forms of chromoblastomycosis. Microbes Infect..

[bib18] Robinson M.J., Osorio F., Rosas M., Freitas R.P., Schweighoffer E., Gross O., Verbeek J.S., Ruland J., Tybulewicz V., Brown G.D. (2009). Dectin-2 is a Syk-coupled pattern recognition receptor crucial for Th17 responses to fungal infection. J. Exp. Med..

[bib19] Rogers N.C., Slack E.C., Edwards A.D., Nolte M.A., Schulz O., Schweighoffer E., Williams D.L., Gordon S., Tybulewicz V.L., Brown G.D., Reis e Sousa C. (2005). Syk-dependent cytokine induction by Dectin-1 reveals a novel pattern recognition pathway for C type lectins. Immunity.

[bib20] Rosas M., Liddiard K., Kimberg M., Faro-Trindade I., McDonald J.U., Williams D.L., Brown G.D., Taylor P.R. (2008). The induction of inflammation by dectin-1 in vivo is dependent on myeloid cell programming and the progression of phagocytosis. J. Immunol..

[bib21] Santos A.L., Palmeira V.F., Rozental S., Kneipp L.F., Nimrichter L., Alviano D.S., Rodrigues M.L., Alviano C.S. (2007). Biology and pathogenesis of Fonsecaea pedrosoi, the major etiologic agent of chromoblastomycosis. FEMS Microbiol. Rev..

[bib22] Takai T., Li M., Sylvestre D., Clynes R., Ravetch J.V. (1994). FcR gamma chain deletion results in pleiotrophic effector cell defects. Cell.

[bib23] Taylor P.R., Tsoni S.V., Willment J.A., Dennehy K.M., Rosas M., Findon H., Haynes K., Steele C., Botto M., Gordon S., Brown G.D. (2007). Dectin-1 is required for beta-glucan recognition and control of fungal infection. Nat. Immunol..

[bib24] Tsoni S.V., Kerrigan A.M., Marakalala M.J., Srinivasan N., Duffield M., Taylor P.R., Botto M., Steele C., Brown G.D. (2009). Complement C3 plays an essential role in the control of opportunistic fungal infections. Infect. Immun..

[bib25] Willment J.A., Lin H.H., Reid D.M., Taylor P.R., Williams D.L., Wong S.Y., Gordon S., Brown G.D. (2003). Dectin-1 expression and function are enhanced on alternatively activated and GM-CSF-treated macrophages and are negatively regulated by IL-10, dexamethasone, and lipopolysaccharide. J. Immunol..

[bib26] Yamasaki S., Matsumoto M., Takeuchi O., Matsuzawa T., Ishikawa E., Sakuma M., Tateno H., Uno J., Hirabayashi J., Mikami Y. (2009). C-type lectin Mincle is an activating receptor for pathogenic fungus, Malassezia. Proc. Natl. Acad. Sci. USA.

