# Supplementary material for: Restoration of Pattern Recognition Receptor Costimulation to Treat Chromoblastomycosis, a Chronic Fungal Infection of the Skin
Source: Cell Host Microbe. 2011 May 19;9(5):436–43. doi: 10.1016/j.chom.2011.04.005 (PMC3098964; doi:10.1016/j.chom.2011.04.005)
Supplement: Document S1. Four Figures [file mmc1.pdf]

Supplemental Information

Cell Host & Microbe, Volume *9*

Restoration of Pattern Recognition Receptor Costimulation to Treat Chromoblastomycosis, a Chronic Fungal Infection of the Skin

Maria da Glória Sousa, Delyth M. Reid, Edina Schweighoffer, Victor Tybulewicz, Jürgen Ruland, Jean Langhorne, Sho Yamasaki, Philip R. Taylor, Sandro R. Almeida, and Gordon D. Brown

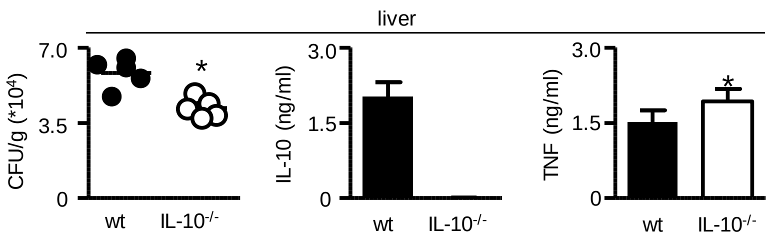

Figure S1, Related to Figure 1. Characterization of Infection with *F. pedrosoi* in IL-10$^{-/-}$ Mice

Fungal burdens and cytokine levels in the livers of infected wild type (black symbols) versus IL-10-/- mice (white symbols). Shown are data ± SD. *; p<0.05.

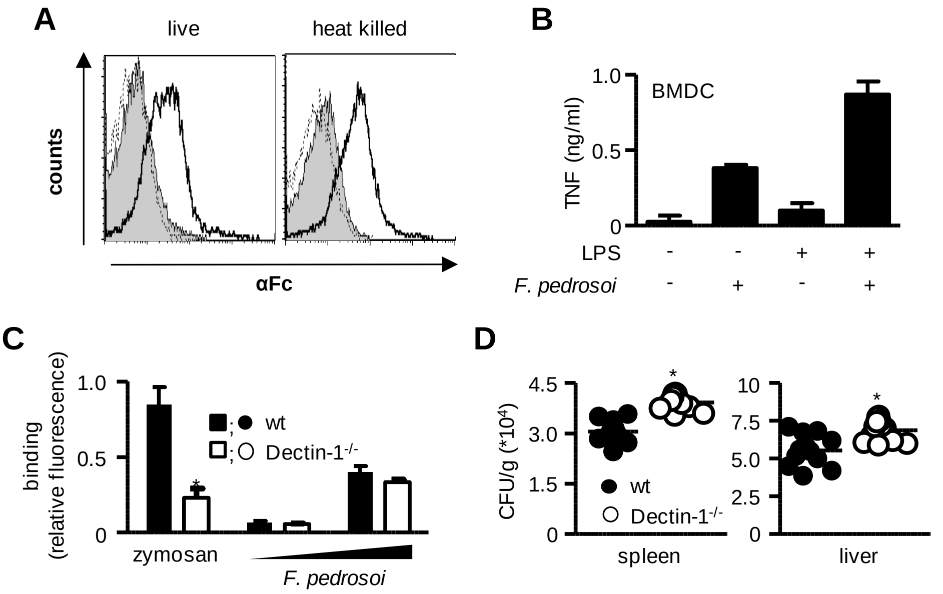

Figure S2, Related to Figure 2. The Role of Dectin-1 in the Response to *F. pedrosoi*

(A) Flow cytometric analysis of exposed β-glucans on live and heat killed *F. pedrosoi* conidia. The fungal particles were probed with Fc-Dectin-1 (black solid line) or Fc-CLEC9A (grey filled histogram). For some samples, to show specificity, Fc-Dectin-1 was pre-treated with soluble β-glucans prior to the addition of conidia (dashed line).
(B) Co-stimulation of murine bone-marrow derived dendritic cells (BMDC) with *F. pedrosoi* (MOI 5:1) or LPS (1ng/ml), as indicated. Shown are the mean ± SD
(C) Binding of zymosan or *F. pedrosoi* conidia (MOI 1:5 and 1:50) to wild-type (wt; back bars) and Dectin-1-deficient (white bars) thioglycollate-elicited macrophages. Data shown are mean ± SD.
(D) Fungal burdens in the spleen and liver of wild-type (back circles) or Dectin-1 deficient mice (white circles), seven days after infection.

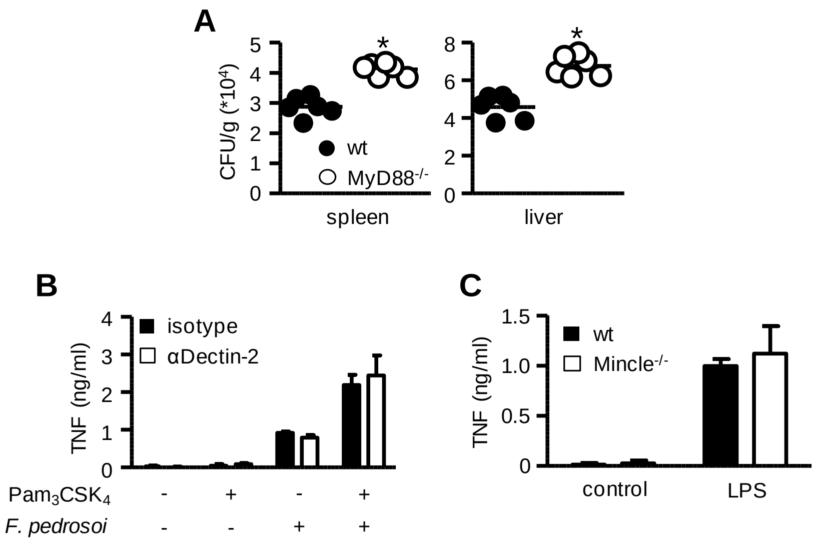

Figure S3, Related to Figure 3. The Role of MyD88, Dectin-2 and Mincle in the Costimulatory Response to *F. pedrosoi*

(A) Fungal burdens in the spleen and liver of wild-type (back circles) or MyD88 deficient mice (white circles), seven days after infection.

(B) TNF levels in the supernatants of BMDC following a three hour stimulation with *F. pedsosoi* or Pam3CSK4, in the presence or absence of inhibitory anti-Dectin-2 antibodies (white bars) or an isotype control (black bars), as indicated. Shown are mean ± SD.

(C) TNF levels in the supernatants of wild-type (wt) or Mincle$^{-/-}$ BMDC (white bars) following a three hour stimulation with LPS (100ng/ml). Shown are mean ± SD.

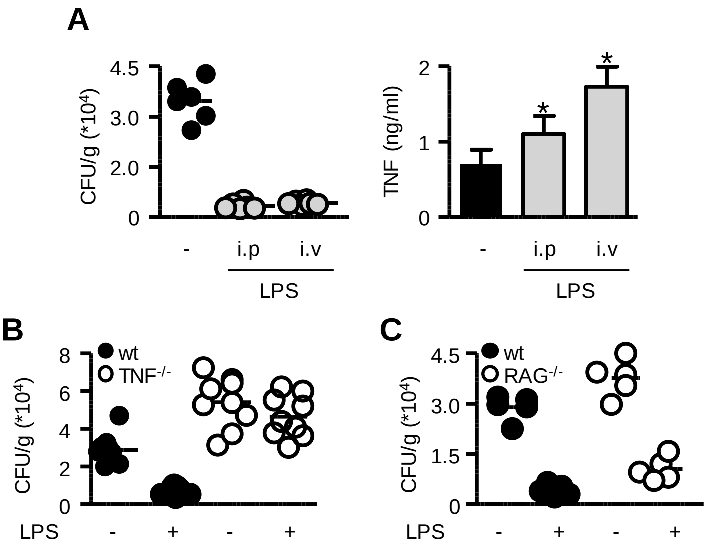

Figure S4, Related to Figure 4. The Effects of TLR Costimulation in vivo

(A) Fungal burdens and TNF levels in the livers of infected mice with (grey symbols) or without (black symbols) LPS treatment, as indicated. Shown are mean ± SD.
(B) Fungal burdens in the livers of infected wild-type (black circles) or $TNF^{-/-}$ mice (white circles) with or without LPS treatment, as indicated.
(C) Fungal burdens in the livers of infected wild-type (black circles) or RAG-/- mice (white circles) with or without LPS treatment, as indicated.
